# ROS and DNA repair in spontaneous versus agonist-induced NETosis: Context matters

**DOI:** 10.3389/fimmu.2022.1033815

**Published:** 2022-11-08

**Authors:** Dhia Azzouz, Nades Palaniyar

**Affiliations:** ^1^ Program in Translational Medicine, Peter Gilgan Centre for Research and Learning, The Hospital for Sick Children, Toronto, ON, Canada; ^2^ Faculty of Medicine, University of Toronto, Toronto, ON, Canada; ^3^ Institute of Medical Sciences, Faculty of Medicine, University of Toronto, Toronto, ON, Canada

**Keywords:** neutrophil extracellular traps (NETs), spontaneous NETosis, reactive oxygen species (ROS), NADPH oxidase (NOX), DNA repair, base excision repair (BER), proliferating cell nuclear antigen (PCNA), DNA polymerases

## Abstract

Reactive oxygen species (ROS) is essential for neutrophil extracellular trap formation (NETosis). Nevertheless, how ROS induces NETosis at baseline and during neutrophil activation is unknown. Although neutrophils carry DNA transcription, replication and repair machineries, their relevance in the short-lived mature neutrophils that carry pre-synthesized proteins has remained a mystery for decades. Our recent studies show that (i) NETosis-inducing agonists promote NETosis-specific kinase activation, genome-wide transcription that helps to decondense chromatin, and (ii) excess ROS produced by NADPH oxidase activating agonists generate genome-wide 8-oxy-guanine (8-OG), and the initial steps of DNA repair are needed to decondense chromatin in these cells. These steps require DNA repair proteins necessary for the assembly and nicking at the damaged DNA sites (poly ADP ribose polymerase PARP, apurinic endonuclease APE1 and DNA ligase), but not the enzymes that mediate the repair DNA synthesis (Proliferating cell nuclear antigen (PCNA) and DNA Polymerases). In this study, we show that (i) similar to agonist-induced NETosis, inhibition of early steps of oxidative DNA damage repair proteins suppresses spontaneous NETosis, but (ii) the inhibition of late stage repair proteins DNA polymerases and PCNA drastically promotes baseline NETosis. Hence, in the absence of excessive ROS generation and neutrophil activation, DNA repair mediated by PCNA and DNA polymerases is essential to prevent chromatin decondensation and spontaneous NETosis. These findings indicate that ROS, oxidative DNA damage, transcription and DNA repair differentially regulate spontaneous and agonist-induced NETosis. Therefore, context matters.

## Introduction

NETosis is the process by which neutrophils release their DNA extracellularly in the form of neutrophils extracellular traps (NETs). This form of cell death is induced by various stimuli, such as phorbol 12-myristate 13-acetate (PMA), lipopolysaccharide (LPS) and *Pseudomonas aeruginosa* (1–7). Douda et al. and others showed that NADPH-oxidase (NOX)-dependent and -independent NETosis, differentially activate various kinases including mitogen-activated protein kinases (extracellular signal-regulated kinase (ERK), p38, c-Jun N-terminal kinase (JNK)) (8–16) in these neutrophils. Khan and Palaniyar later showed that activated kinase cascades facilitate genome-wide transcriptional activation that is necessary for chromatin decondensation and NET release, regardless of the type of NETosis (17). NETs are released as chromatin decorated with cytotoxic peptides and enzymes, including LL-37, myeloperoxidase (MPO) and several neutrophil proteases (1–7, 18).

During NOX-dependent NETosis, activation of NOX generates large amounts of ROS. We recently showed that oxidative DNA damage repair is also instrumental in the ability of activated neutrophils to undergo NETosis (19). We uncovered that NOX activation in neutrophils results in significant oxidative DNA damage, followed by the translocation of proliferating cell nuclear antigen (PCNA), a key DNA repair protein stored in the cytoplasm into the nucleus. Furthermore, in these highly activated neutrophils, inhibiting key DNA repair proteins (poly ADP ribose polymerase PARP, apurinic endonuclease APE1 and DNA ligase) drastically reduces the ability of neutrophils to undergo NETosis.

Baseline levels of ROS are known to be produced in neutrophils and necessary for spontaneous NETosis (20–22). Hence, we determined the role of ROS and base excision repair (BER) machinery in spontaneous NETosis. These studies show that DNA repair synthesis steps are essential to prevent spontaneous NETosis, and inhibition of DNA repair polymerases and PCNA promotes spontaneous NETosis. Collectively, different steps of the DNA repair pathway differentially manifest in NETosis at baseline and during activation, and hence, the context matters.

## Methods

### Ethical clearance and samples isolation

Protocols were approved by the Hospital for Sick Children research ethics committee. Peripheral blood was drawn from healthy donors. Isolation was performed as previously described (4). Briefly, neutrophils were isolated using PolymorphPrep (Axis-Shield). Red blood cells were lysed using a hypotonic solution of 0.2% (w/v) NaCl. Neutrophils were resuspended in RPMI-1640 medium (Invitrogen) for experiments.

### SYTOX Green plate reader assay for NETosis analysis

SYTOX Green dye (5 μM; ThermoFisher Scientific) was added to cells (5×10^5^ cells/ml). A total of 50,000 cells were seeded per well on a 96-well plate. PMA (25 nM) was used as an agonist to induce NOX-dependent NETosis (positive control). ROS inhibitors (NOX inh Diphenyleneiodonium chloride, 1 μM DPI, Sigma), ROS scavengers (N-Acetyl-L-cysteine, 3 mM NAC, Sigma) and fetal bovine serum (1% (v/v) FBS, ThermoFisher Scientific) were added to the cells 1 hour before adding DNA repair inhibitors: Pol δ inh (Aphidicolin, 50 μM, Sigma), Pol β inh (AM-TS23, 25 μM, Tocris), PCNA : Polymerase interaction inh or PCNA inh (T2AA, 25 μM, Tocris), APE inh 1 (CRT0044876, 125 μM, Sigma), APE inh 2 (APE1 Inhibitor III, 50 μM, EMD-Millipore), PARP1 inh 1 (BSI201, 100 μM, Sigma), PARP inh 2 (PJ34, 50 μM, EMD-Millipore), LIG inh (L189, 100 μM, Tocris). Measurements were taken as soon as the DNA repair inhibitors were added. Fluorescence of SYTOX Green-DNA interaction was measured using POLARstar OMEGA fluorescence plate reader (BMG Labtech; 485nm/525nm) at 4 hours. NETosis index was determined by dividing the fluorescence reading of each treatment by the reading of 1% (v/v) Triton X-100-treated cells. Plotted data represent NETosis levels.

### DHR123 plate reader assay

Cells (1×10^6^ cells/ml) were seeded on a 96-well plate at a volume of 100 μl. Dihydrorhodamine-123 (DHR123, 25 µM) was added to cells for 30 minutes before being treated with media, PMA or DNA repair inhibitors. Florescence of DHR123 oxidation was measured using POLARstar OMEGA fluorescence plate reader (BMG Labtech; 485nm/525nm) after 4 hours.

### Confocal imaging

Cells (1×10^6^ cells per ml) were plated on a 96-well plate, and incubated with ROS inhibitors for 1 hour at 37°C. After adding Pol δ inh, Pol β inh or PCNA:polymerase interaction inh, NETosis was allowed to proceed for 4 hours at 37°C before being terminated with 4% (w/v) paraformaldehyde (Sigma) overnight. Cells were permeabilized with 1% (v/v) Triton X-100 for 25 minutes and then blocked with 2.5% (w/v) BSA in PBS for 1 hour. PCNA was probed for using mouse anti-PCNA antibody (F-2, Santa Cruz) at a 1:250 dilution. MPO was probed using mouse anti-MPO antibody (ab25989, Abcam) at a 1:500 dilution. DAPI (10 μM; ThermoFisher Scientific) at 1:333 dilution was used for visualising DNA. Imaging was done using Olympus IX81 inverted fluorescence microscope with a Hamamatsu C9100-13 back-thinned EM-CCD camera and Yokogawa CSU×1 spinning disk confocal scan head.

### Statistical analyses

Statistical analyses were performed using GraphPad Prism 7. One-Way ANOVA with Dunnett and Tukey’s post-tests or Two-way ANOVA with Dunnett’s post-test were performed as appropriate. Variance between groups compared is similar. Error bars in graphs represent ± SEM. A p-value <0.05 was considered to be statistically significant.

## Results

### ROS inhibitors inhibit spontaneous NETosis

**Figure 1:**
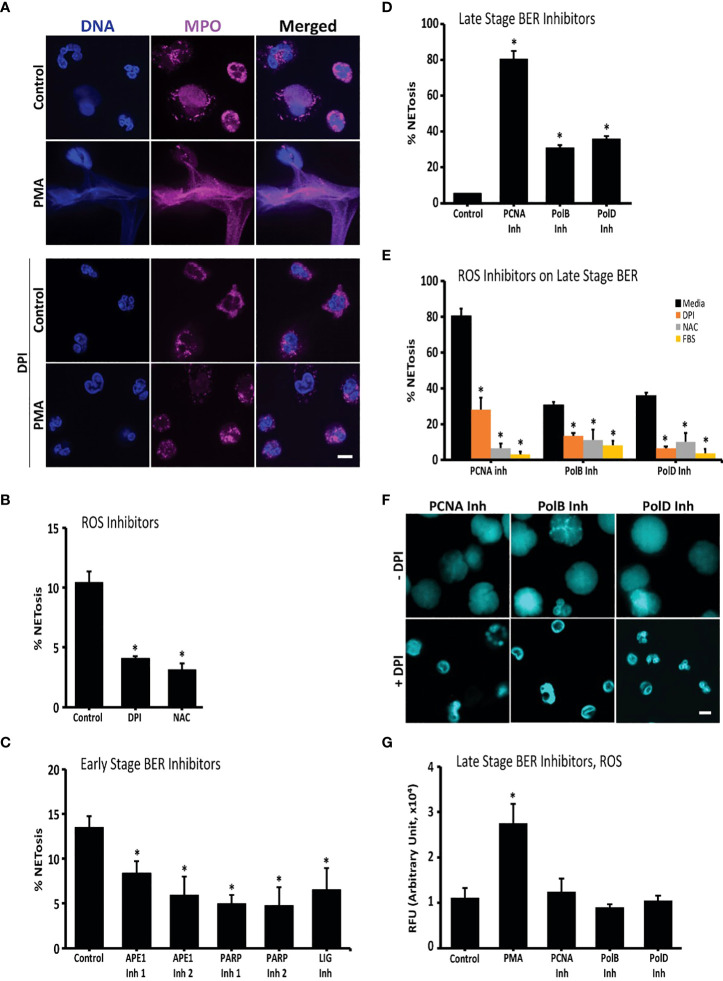
Oxidative DNA damage and repair drive NETosis: inhibition of early repair steps suppresses spontaneous NETosis while inhibition of late repair steps promotes it. **(A)** Immunoconfocal imaging confirms that neutrophils undergo low level spontaneous NETosis in media, while PMA, an oxidative burst inducer, promotes robust NETosis. Presence of DPI, an inhibitor of ROS generation, suppresses NETosis in both experimental conditions. DNA (DAPI, blue), MPO (pink). **(B)** NET DNA release from neutrophils incubated in media, or media with DPI or NAC indicates that ROS inhibitors suppress spontaneous NETosis (SYTOX; *, p<0.05 compared to control). **(C)** DNA release from neutrophils incubated in media or media with inhibitors (inh) of early stage oxidative DNA damage repair proteins APE (inh 1 or 2), PARP1 (inh 1 or 2) or LIG suggests that inhibition of these proteins suppresses spontaneous NETosis (SYTOX; *, p<0.05 compared to control). **(D)** DNA release from neutrophils incubated in media, or media with inhibitors of late stage repair proteins PCNA, Pol β or Pol δ suggest that inhibition of these proteins promotes spontaneous NETosis (SYTOX; *, p<0.05 compared to control). **(E)** DNA release from neutrophils incubated with ROS inhibitors (DPI, NAC or FBS) shows that spontaneous NETosis is suppressed by ROS inhibitors regardless of the presence of inhibitors to PCNA, Pol β or Pol δ in the media (SYTOX; *, p<0.05 compared to media treated control in each respective cluster). **(F)** Confocal imaging confirms that ROS inhibitor DPI suppresses spontaneous NETosis promoted by the inhibitors of PCNA, Pol β or Pol δ. DNA (DAPI, blue). **(G)** ROS measurements by DHR123 assays show that neutrophils used in the experiments generate high levels of ROS when stimulated with PMA (+ve control) compared to non-activated neutrophils. Presence of inhibitors to PCNA, Pol β or Pol δ does not alter the baseline ROS generation (Plate reader; *, p<0.05 compared to control). Serum was not added to the media unless otherwise stated. DNA repair inhibitors used were: APE inh 1 (CRT0044876), APE inh 2 (APE1 Inhibitor III), PARP1 inh 1 (BSI201), PARP inh 2 (PJ34) or LIG inh (L189), PCNA inh (T2AA), Pol β inh (AM-TS23) or Pol δ inh (Aphidicolin). Fluorescence was recorded at 4-hour time points by SYTOX Green plate reader assays (n = 3 for all the experiments). The images are representative of 3 independent experiments, at 4-hour time points. Scale bar on images, 5 μm.

NETosis in healthy neutrophils and neutrophils treated with PMA (with and without DPI) was imaged for MPO colocalization to NET DNA to confirm whether the neutrophils were capable of undergoing NOX-dependent NETosis. Most neutrophils in the PMA condition underwent NETosis, while a small minority of the unstimulated cells had undergone NETosis (**Figure 1A**). NOX inhibitor, DPI, inhibited both spontaneous and agonist-induced NETosis (**Figure 1A**).

To determine whether endogenous ROS induced the observed background NETosis, NETosis levels of untreated neutrophils were compared to levels of neutrophils treated with ROS inhibitors. NOX inhibitor, DPI, and the general ROS scavenger, NAC, were used. Untreated neutrophils were observed to undergo NETosis, termed background or spontaneous NETosis, as determined by the SYTOX Green plate reader assays (**Figure 1B**). SYTOX Green is a cell impermeable dye that fluoresces green when it binds to extracellular DNA, such as NETs. These assays showed that inhibiting ROS production and scavenging ROS suppressed spontaneous NETosis. DPI suppressed background NETosis, confirming that spontaneous NETosis is the result of endogenous ROS produced by NOX (**Figures 1A, B**).

### Inhibitors of later steps of DNA repair induce NETosis while inhibitors of early steps suppress spontaneous NETosis

To elucidate the role of DNA repair in background NETosis, inhibitors against DNA repair machinery proteins were used and their effects were studied using the SYTOX Green plate reader assays and immunoconfocal microscopy. First, cells were incubated with BER pathway inhibitors (APE1, PARP1, DNA ligase, PCNA and polymerase β/δ inhibitors) and NETosis levels were determined after 4 hours by SYTOX assays. Interestingly, PCNA and polymerase β/δ inhibitors induced NETosis while APE1, PARP1 and DNA ligase inhibitors reduced background NETosis levels (**Figures 1C, D**). As determined by DHR123 assays, APE1, PARP1 and DNA ligase inhibitors did not affect ROS levels in neutrophils, which indicates that their NETosis-inhibiting effect does not involve reduction in endogenous ROS levels (**Figure S1**).

As ROS and DNA repair are interconnected, the role of ROS in the induction of NETosis by PCNA and polymerase β/δ inhibitors was studied. We used three ROS inhibitors to determine the role of ROS in the process. DPI, NAC and FBS were all found to inhibit NETosis induced by PCNA and polymerase β/δ inhibitors (**Figures 1E, F**; **S2**). To verify that PCNA and polymerase β/δ inhibitors were not inducing NETosis by increasing ROS production, we studied ROS levels using DHR123 assay. We found that these late stage BER inhibitors did not affect ROS levels in neutrophils (**Figure 1G**). We also confirmed that the presence of PCNA inhibitor did not alter the effect of ROS inhibitors. Pre-treating neutrophils with the ROS inhibitors DPI, NAC and FBS inhibited spontaneous ROS production even in the presence of PCNA inhibitors (**Figure S3**). Hence, baseline oxidative DNA damage induces spontaneous NETosis, and inhibiting late stage BER proteins promote spontaneous NETosis.

**Figure 2:**
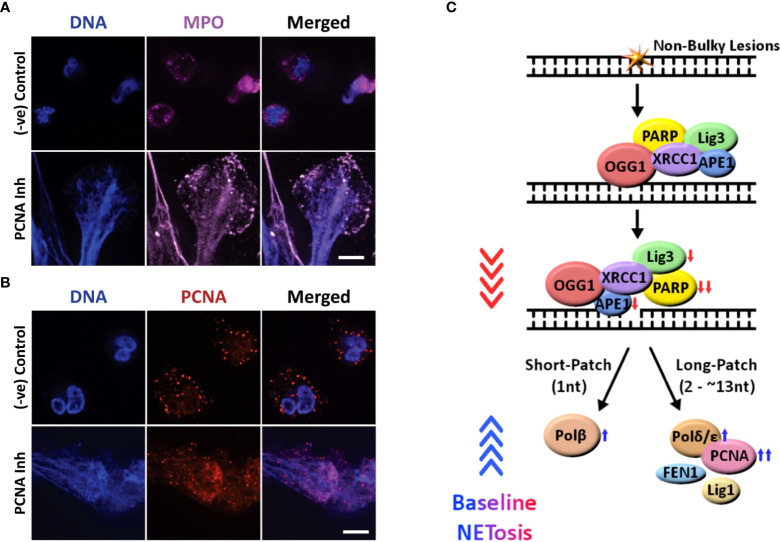
Confocal images showing that inhibiting the late stage oxidative DNA damage repair pathways induce spontaneous NETosis, and a diagram summarizing the regulation of spontaneous NETosis by various DNA repair proteins. **(A, B)** Neutrophils were treated with PCNA inhibitor (T2AA), immunostained and imaged by confocal microscopy. MPO (pink) colocalizing to DNA (DAPI blue) shows that PCNA inhibition promotes spontaneous NETosis **(A)**. PCNA (red) is cytoplasmically distributed in the neutrophils but the inhibition of late stage DNA repair by inhibition of PCNA: polymerase binding leads to NETosis with PCNA present throughout the NET DNA (DAPI, blue) **(B)**. Images for panels are representative of 3 independent experiments. Scale bar, 10 μm. **(C)** Diagram highlighting the proteins involved in the oxidative DNA damage repair pathways and their effects on spontaneous NETosis. Non-bulky adducts are repaired by BER. Baseline NETosis is suppressed by early DNA repair events that lead up to the chromatin decondensation/nick formation by APE1. Inhibition of late events of DNA repair promotes spontaneous NETosis. Proteins whose inhibition induces (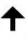, blue arrow) or suppresses (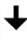, red arrow) spontaneous NETosis are indicated. Overall, inhibition of early and late steps of oxidative DNA damage repair pathway result in powerful inhibition (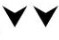, red arrowheads) and activation (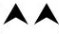 blue arrowheads) of spontaneous NETosis, respectively.

One of the key markers of NETosis is MPO colocalization to the extracellularly released DNA. To confirm that polymerase inhibition results in NETosis, and not another form of cell death such as necrosis, we imaged for MPO. We found that this NET marker colocalized to decondensed chromatin, confirming that inhibiting PCNA:polymerase interaction (using T2AA) induces NETosis (**Figures 2A**, **S4**). We have previously shown that PCNA is found throughout NETs during agonist-induced NETosis. To confirm the role of DNA repair in NETosis promoting role of PCNA inhibitor, we immunoimaged PCNA (23). We found PCNA to be colocalized on the NETs in cells treated with the inhibitor (while being primarily localized to the cytoplasm in untreated cells) which supports a role of DNA repair pathways during PCNA:polymerase interaction inhibitor in promoting baseline NETosis (**Figure 2B**). These results indicate that PCNA/polymerase inhibitors induce NETosis through their inhibition of the final stages of the DNA repair pathway (**Figure 2C**).

## Discussion

ROS induces DNA lesions in the form of modified bases. The lesions halt transcription and DNA replication. While neutrophils do not replicate their DNA, they have evolved to repair DNA damage (24). We have previously shown that transcription is important for NETosis (17). Neutrophils are known to carry many DNA repair proteins, including OGG1, PCNA, PARP, and DNA pol β (25–28). We have shown for the first time that background NETosis in healthy neutrophils is driven by ROS and DNA repair (**Figure 2C**). Neutrophils were found to undergo background NETosis. The observed NETosis was suppressed when the cells were treated with a NOX inhibitor (DPI) or a ROS scavenger (NAC). This indicated that the background NETosis observed in untreated healthy neutrophils was caused by the endogenously produced ROS. Inhibitors of the steps of DNA repair that occur post-chromatin unwinding (PCNA and polymerase β/δ inhibitors) led to increased NETosis levels while inhibitors of earlier steps inhibited NETosis. Inhibiting one of the polymerases resulted in only partial NETosis, while inhibiting PCNA interaction with polymerases resulted in much higher levels of NETosis (**Figure 1E**). This suggests that PolB and PolD are both involved in repairing DNA damaged caused by endogenous ROS and participate in regulating NETosis.

During DNA repair, after the addition of the new base and ligation of the strand, machinery falls off the newly repaired site and the chromatin is rewound. One postulation to account for the observed results is that inhibition of steps that follow unwinding result in a stage of permanently unwound chromatin at the site of damage as the damage does not get repaired. As the DNA continues to sustain oxidative damage from the endogenously produced ROS, sites of permanently unwound chromatin accumulate and are the driving force of the chromatin unwinding that leads to the neutrophils undergoing NETosis. Inhibitors of the early steps of DNA repair reduced NETosis because they inhibit steps that occur before the unwinding (29) and, as a result, prevent the formation of sites of temporarily and permanently unwound chromatin.

A potential explanation for the results observed is the unwinding capabilities of DNA repair machinery (30). We observed that inhibition of steps that prevent the assembly of early proteins of BER machinery, which includes APE1, at the site of damage prevented baseline NETosis (31). This suggests that inhibition of nicking of the DNA by APE1 results in the loss of neutrophils’ ability to undergo NETosis. Hence, it is possible that it is the DNA nicking capabilities of DNA repair machinery that is mainly responsible for driving chromatin decondensation for NETosis in neutrophils.

We observed that like in agonist-induced NETosis (23) (induced by PMA and LPS), BER plays a significant contribution in spontaneous NETosis (**Figures 1**, **2**). In the absence of any agonists, cells produce limited amounts of ROS (32) and it is expected that single adduct formations are the primary type of oxidative DNA damage (33). Therefore, it is expected that BER would play a significant role. However, during activated NETosis, much higher levels of ROS production are observed (23, 34). Hence, there is increased chance of forming bulky adducts which would require repair by nucleotide excision repair (NER) (35). Due to the large role transcription plays in active NETosis (17), both global genome NER (GG-NER) and transcription coupled NER (TC-NER) could be responsible for the repair of bulky lesions. We expect the exact contributions of BER and GG-/TC-NER to depend on the type of agonist, and the type of ROS produced since that would affect the type of oxidative damage the DNA sustains.

One of the key findings is that NETosis outcome when neutrophils were treated with DNA repair inhibitors relied on the context. In activated neutrophils (treated PMA and LPS), PCNA and polymerase inhibitors did not reduce NETosis (23). However, in the case of untreated neutrophils, PCNA and polymerase inhibitors drastically increased spontaneous NETosis (confirmed by studying MPO-NET colocalization). One explanation is that in the case of spontaneous NETosis, the rate of oxidative DNA damage is low, and the DNA Polymerase and PCNA present can sufficiently complete the repair and allow for the repair machinery to fall off and the chromatin to be rewound. Hence, when PCNA and polymerases are inhibited, the nick in the DNA created by APE1 at the site of DNA damage is not repaired and, as a result, the chromatin remains unwound. However, during activated NETosis, the rate of oxidative damage occurring far exceeds the ability of PCNA and polymerase to manage all sites of repair; hence, regardless of whether PCNA and polymerase are inhibited or not, NETosis will occur. This could explain why the activated neutrophils will continue to undergo NETosis unchanged when treated with PCNA and polymerase inhibitors (19).

Another example of how context matters is the differential effect of the host environment on neutrophils’ ability to undergo NETosis (20–22). In a high antioxidant environment, such as in the presence of serum in blood, which contains albumin, we observe reduced NETosis (**Figure 1B**). However, when the neutrophils migrate to environments with limited antioxidants, such as in tissues, neutrophils respond differently and are more susceptible to NETosis (**Figure 1A**). As we saw from our results, in a low antioxidant environment (media with no FBS supplement, akin to extravascular spaces), PCNA and polymerase inhibitors induced NETosis (**Figure 1D**). However, in a high antioxidant environment (media with FBS, akin to intravascular blood), the neutrophils did not undergo NETosis in the presence of the PCNA and polymerase inhibitors (**Figure 1E**). Therefore, depending on the context, NETosis regulation differs and the pathways behave differently.

Inhibition of ROS using DPI, NAC and FBS led to reduction in NETosis induced by PCNA and polymerase β/δ inhibitors. This indicates that the process involves ROS and, therefore, the DNA repair affecting abilities of the drugs. The finding that inhibitors of DNA repair steps post chromatin unwinding at the site of damage suggest that it is only the chromatin unwinding capability of the DNA repair machinery that drive background NETosis and not the repair of the site itself. In summary, by oxidising DNA bases, ROS is recruiting the lesion detection and chromatin remodeling complexes of the DNA repair pathway. Hence, we have identified a unique phenomenon that when any DNA repair pathway component after the unwinding/nicking step is suppressed, neutrophils undergo spontaneous NETosis.

Findings of these studies have great potential implications in disease states and SNPs where certain DNA repair proteins are less active or less expressed. Furthermore, mutations in such DNA repair genes may play a role in inflammatory and autoimmune diseases such as lupus and arthritis. For instance, a POLB mouse model where PolB is less efficient has been reported to exhibit lupus symptoms. It is possible that the observed phenotype may be the result of excess NETosis (which have been implicated in Lupus (36–39)) as a result of the mutation in PolB, and other repair proteins and enzymes (40).

## Data availability statement

The raw data supporting the conclusions of this article will be made available by the authors, without undue reservation.

## Ethics statement

The studies involving human participants were reviewed and approved by Hospital for Sick Children Ethics Committee. The patients/participants provided their written informed consent to participate in this study.

## Author contributions

DA conducted experiments, analyzed the data, generated figures and drafted the manuscript. NP is the principal investigator, obtained funding for the study, conceived the idea, designed the study, edited the manuscript and supervised the research project. All authors contributed to the article and approved the submitted version.

## Funding

DA is a recipient of OTOSF/Restracomp studentship, Ontario Graduate Scholarship and University of Toronto Fellowship. This study was supported by research grants of Canadian Institutes of Health Research (MOP-111012 to NP), and Natural Sciences and Engineering Research Council of Canada (RGPIN436250-13 to NP).

## Conflict of interest

The authors declare that the research was conducted in the absence of any commercial or financial relationships that could be construed as a potential conflict of interest.

## Publisher’s note

All claims expressed in this article are solely those of the authors and do not necessarily represent those of their affiliated organizations, or those of the publisher, the editors and the reviewers. Any product that may be evaluated in this article, or claim that may be made by its manufacturer, is not guaranteed or endorsed by the publisher.
