# Supplementary material for: ROS and DNA repair in spontaneous versus agonist-induced NETosis: Context matters
Source: Front Immunol. 2022 Nov 8;13:1033815. doi: 10.3389/fimmu.2022.1033815 (PMC9679651; doi:10.3389/fimmu.2022.1033815)
Supplement: Supplementary file 1 [file DataSheet_1.pdf]

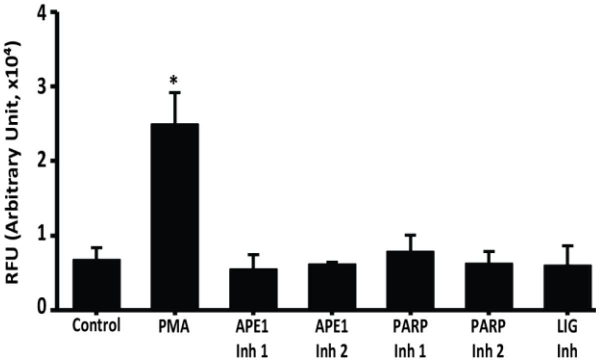

Figure S1 | Inhibitors to early oxidative DNA damage repair proteins do not affect ROS levels. Neutrophils were incubated with DHR123, incubated in media, or media with an oxidative burst inducer PMA (+ve control), or inhibitors for DNA repair pathway proteins APE (inh 1, CRT0044876; inh 2, APE1 Inhibitor III), PARP1 (inh 1, BSI201; inh 2, PJ34) or LIG (L189). The probe oxidised by ROS was measured by plate reader assays (R123 fluorescence; n = 3; *, $p<0.05$ compared to control).
