# Supplementary material for: ROS and DNA repair in spontaneous versus agonist-induced NETosis: Context matters
Source: Front Immunol. 2022 Nov 8;13:1033815. doi: 10.3389/fimmu.2022.1033815 (PMC9679651; doi:10.3389/fimmu.2022.1033815)
Supplement: Supplementary file 2 [file DataSheet_2.pdf]

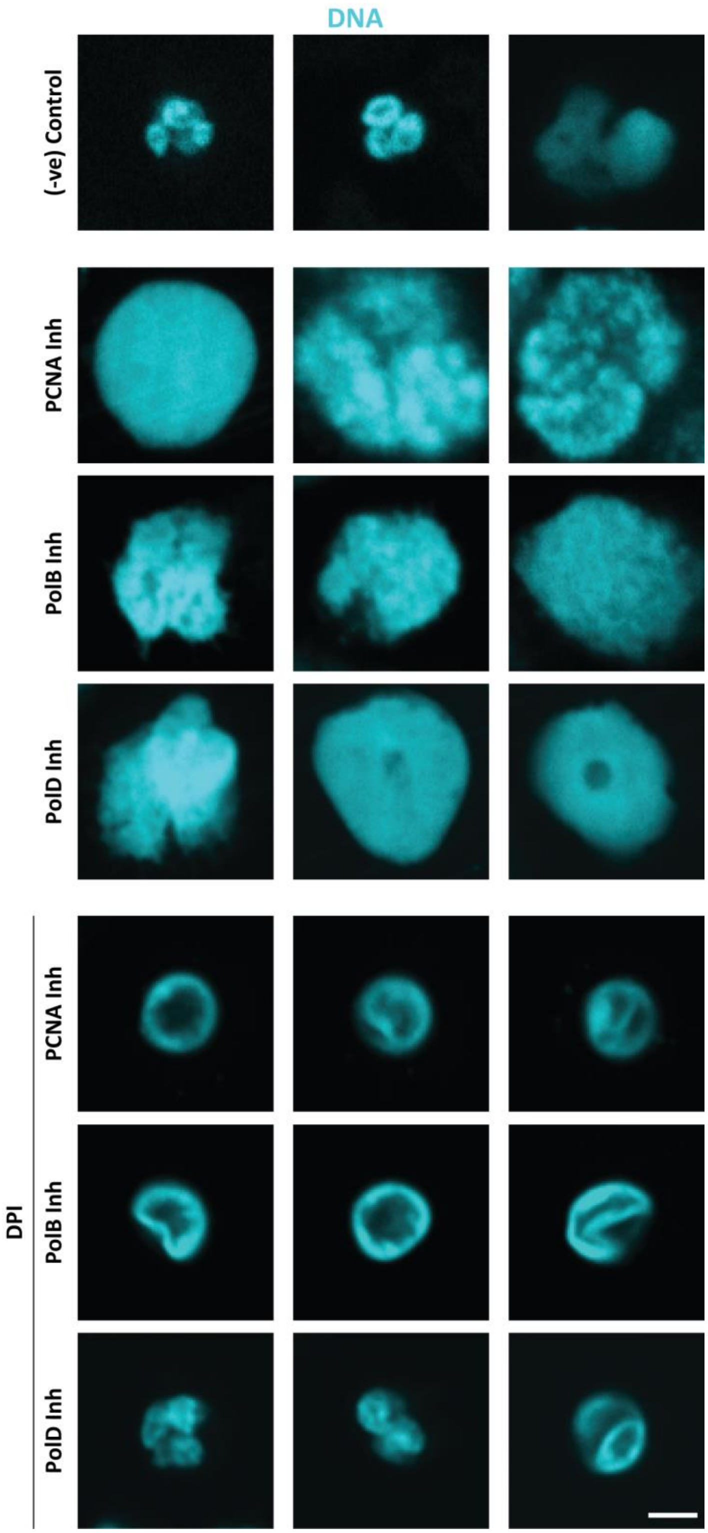

Figure S2 | Single cell confocal images of a ROS inhibitor, DPI, reducing PCNA- and DNA polymerase-induced baseline NETosis. Neutrophils were incubated in media with or without DPI for 1 hour, treated with inhibitors (inh) for PCNA (T2AA), Pol β (AM-TS23) or Pol δ (Aphidicolin) for 4 hours, and stained for DNA (DAPI, blue). Three example images for each condition are representative of 3 independent experiments. Scale bar, 5 µm.
