# Supplementary material for: ROS and DNA repair in spontaneous versus agonist-induced NETosis: Context matters
Source: Front Immunol. 2022 Nov 8;13:1033815. doi: 10.3389/fimmu.2022.1033815 (PMC9679651; doi:10.3389/fimmu.2022.1033815)
Supplement: Supplementary file 3 [file DataSheet_3.pdf]

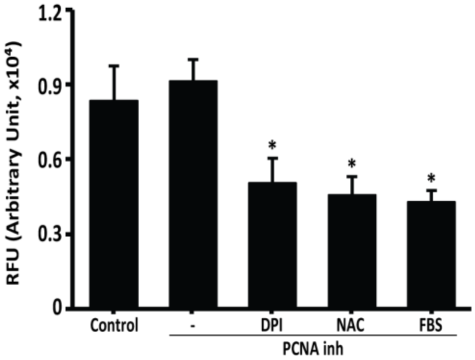

Figure S3 | Regardless of the presence of PCNA inhibitor, ROS inhibitors suppress baseline ROS production in neutrophils. Neutrophils were incubated with media or media with ROS inhibitors (DPI, NAC or FBS), incubated with ROS detection fluorescence probe DHR123 for 1 hour, and then treated with PCNA inhibitor (T2AA). The probe oxidised by ROS was measured by plate reader assays (R123 fluorescence; n = 3; *, p<0.05 compared to media control without any ROS inhibitors).
