# Supplementary material for: ROS and DNA repair in spontaneous versus agonist-induced NETosis: Context matters
Source: Front Immunol. 2022 Nov 8;13:1033815. doi: 10.3389/fimmu.2022.1033815 (PMC9679651; doi:10.3389/fimmu.2022.1033815)
Supplement: Supplementary file 4 [file DataSheet_4.pdf]

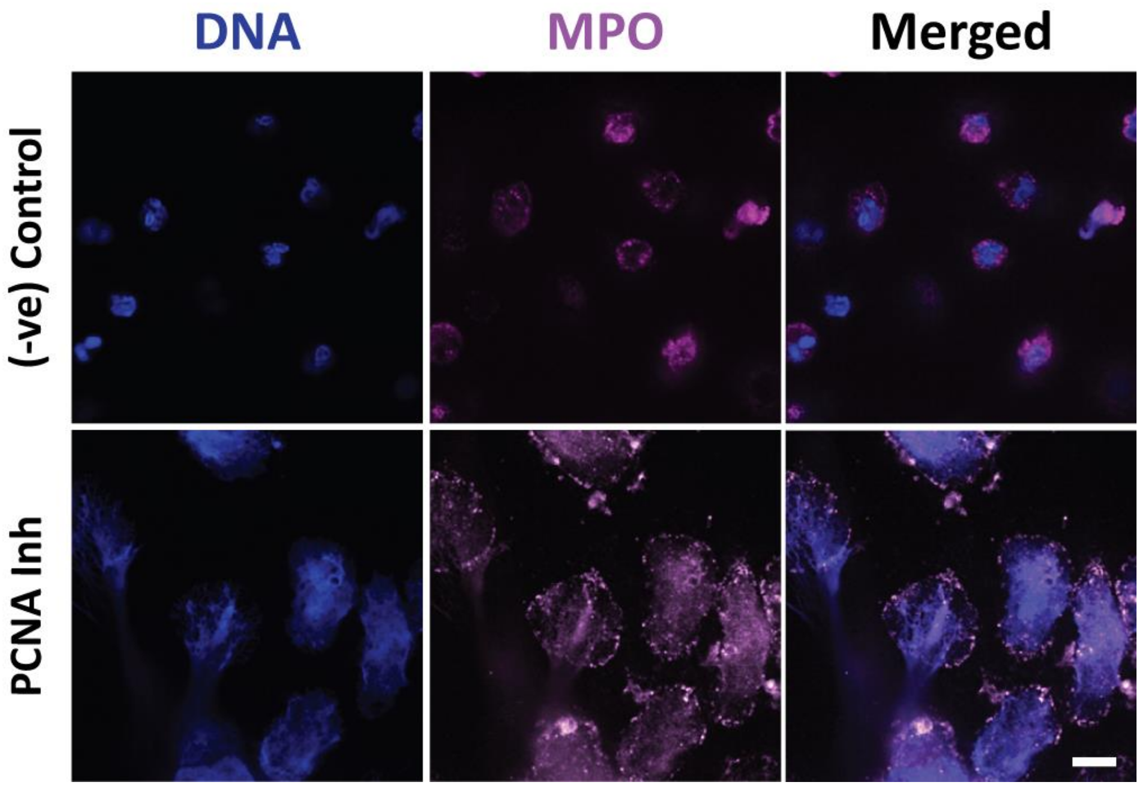

Figure S4 | Low magnification confocal images show that PCNA inhibitors induce spontaneous NETosis. Neutrophils were treated with PCNA inhibitor (T2AA), immunostained and imaged. immunofluorescence imaging shows that MPO (pink) colocalizes to decondensed/extracellular DNA (DAPI, blue); hence T2AA induces spontaneous NETosis in most of the cells. Images are representative of 3 independent experiments. Scale bar, 15 µm.
